# Supplementary material for: Leveraging diverse cell-death patterns in diagnosis of sepsis by integrating bioinformatics and machine learning
Source: PeerJ. 2025 Feb 26;13:e19077. doi: 10.7717/peerj.19077 (PMC11871900; doi:10.7717/peerj.19077)
Supplement: Supplemental Information 2 [file peerj-13-19077-s002.pdf]

Figure S1

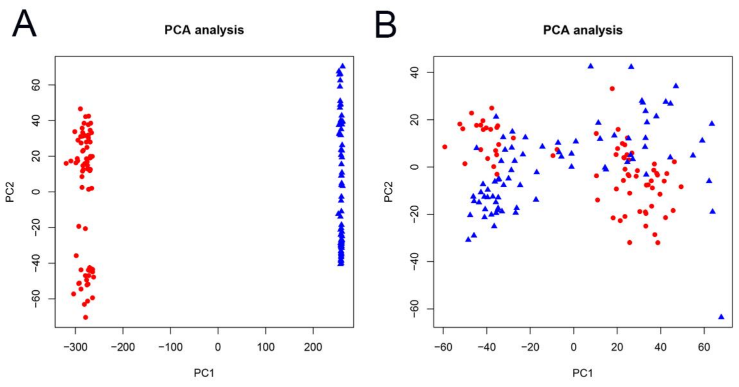

Figure S1. (A) PCA analysis of GSE95233 and GSE154918 before batch-effect correction. (B) PCA analysis of GSE95233 and GSE154918 after batch-effect correction.

Figure S2

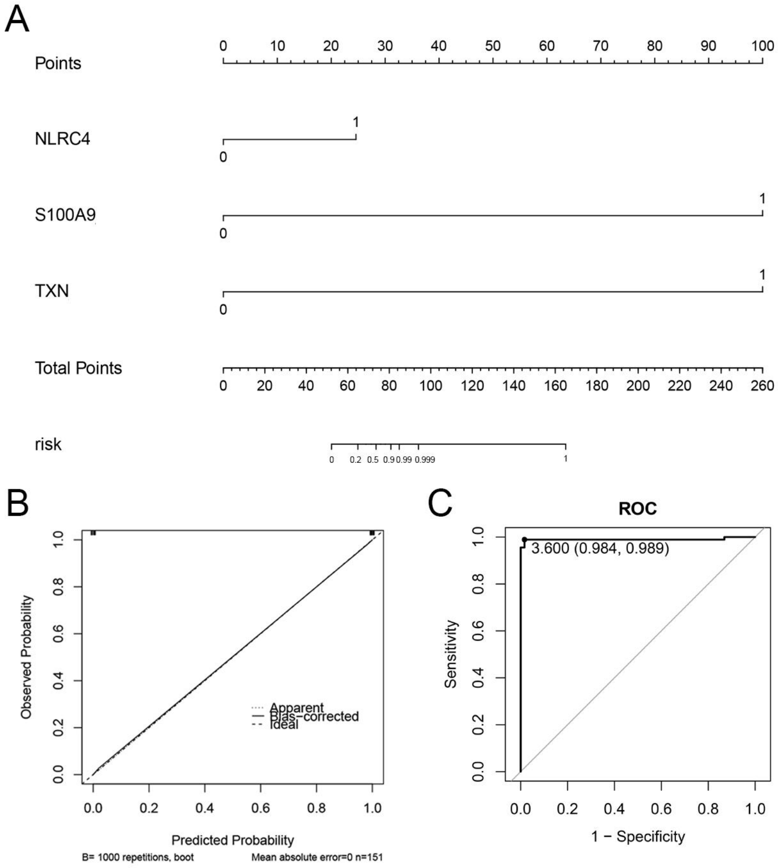

Figure S2. (A) Nomogram constructed for diagnosis of sepsis using cutoff values of biomarkers. (B) Calibration curve for nomogram. (C) The ROC curve of the efficacy of three biomarkers in training set.
